# Massage for gastrointestinal function among participants after abdominal surgery: A protocol for systematic review and meta-analysis

**DOI:** 10.1097/MD.0000000000028087

**Published:** 2021-12-10

**Authors:** Yongliang Wang, Jiaben Xu, Rui Bao, Zhaoxian Li

**Affiliations:** a Heilongjiang University of Chinese Medicine, Harbin, China; b Second Affiliated Hospital, Heilongjiang University of Chinese Medicine, Harbin, China.

**Keywords:** massage, postoperative gastrointestinal dysfunction, protocol, systematic review

## Abstract

**Background:**

Postoperative gastrointestinal dysfunction (PGD) is one of the most common complications among participants undergoing abdominal surgery, with an incidence of 10%–30%. In China, massage is generally the most widely used technique to treat various diseases by the theory of Yin and Yang. In this study, our aim is to assess the effect and safety of massage on gastrointestinal function among participants undergoing abdominal surgery.

**Methods:**

We will search seven databases including Cochrane Library, MEDLINE, EMBASE, CNKI, VIP, CBM and WANGFANG. Meanwhile, we will include all randomized controlled trials if they recruited participants undergoing abdominal surgery. Primary outcomes will be the time to first defecation. Two authors will independently scan all the potential articles, extract the data and assess the risk of bias by Cochrane tool of risk of bias. Al analysis will be performed by RevMan 5.3 software. Dichotomous variables will be expressed as RR with 95% CIs and continuous variables will be reported as MD with 95% CIs. If possible, a fixed or random effects models will be conducted and the confidence of cumulative evidence will be assess using GRADE.

**Results:**

This study will be to assess the effect and safety of massage on gastrointestinal function among participants undergoing abdominal surgery.

**Conclusions:**

This study will assess the effect and safety of massage among participants undergoing abdominal and move forward to help inform clinical decisions.

## 1 Introduction

Postoperative gastrointestinal dysfunction (PGD) is one of the most common complications in participants undergoing abdominal surgery,^[1–3]^ which affects the gastric, intestinal, and biliary tract systems. Clinical symptoms among PGD patients undergoing abdominal surgery include abdominal distention, constipation, nausea and vomiting, and defecation disorders, with an incidence of 10% to 30%.^[4–6]^ The main reasons lead to PGD including age, difference in constitution, method of anesthesia and postoperative analgesia, inflammatory reaction and psychopathology.^[6–10]^

In China, massage, a 3000-year-old ancient system of medicine is generally the most widely used technique to treat various diseases. Massage is a healing system that guided by the theory of Yin and Yang. Recently, clinical practices have proved that there is good curative effect for massage on the gastrointestinal dysfunction by decreasing time to first passage of flatus and time to toleration of diet.^[10–12]^ Its mechanism may be to improve gastrointestinal motility, digestion, absorption, secretion and immune function.

Up to present, there is no published systematic review been conducted to summarize the evidence on the massage for PGD. Therefore, it is of great importance to perform systematic reviews and meta-analyses of the randomized controlled trials (RCTs) on the effects of massage for PGD. In this study, we will conduct a systematic review and meta-analysis of RCTs to evaluate the current evidence on the effects of massage on gastrointestinal function among participants undergoing abdominal surgery and move forward to help inform clinical decisions.

## 2 Methods and analysis

### 2.1 Objectives and registration

This review will be to assess and summarize the available evidence of massage on gastrointestinal function among participants undergoing abdominal surgery. This review protocol is adhere to the preferred reporting items for systematic reviews and meta-analyses statement^[13]^ and registered in the OSF platform (https://osf.io/registries) with a registration number 10.17605/OSF.IO/UCJY6.

### 2.2 Eligibility criteria

#### 2.2.1 Type of study design

We will include all randomized controlled trials involving massage for PGD in this systematic review regardless of publication status and language. Quasi-RCTs and nonrandomized studies will be excluded.

#### 2.2.2 Types of participants

In this study participants undergoing abdominal surgery will be included regardless of their age, or race, surgery type, educational and economic status.

#### 2.2.3 Types of interventions

We will include all types of massage with no limitations of the type of massage, dosage or duration of intervention. In included RCTs comparisons will be massage versus no treatment, placebo or other therapeutic agents.

#### 2.2.4 Types of outcomes

The primary outcome will be the time to first defecation. Secondary outcomes will include the time to first passage of flatus, the time to first bowel movement sound, the time to tolerance of solid food, and adverse events.

### 2.3 Information sources and search strategy

We will search seven electronic databases including Cochrane Library, MEDLINE, EMBASE, Chinese BioMedical Database (CBM), China National Knowledge Infrastructure (CNKI), Chinese VIP Information (VIP) and Wangfang Database regardless of publication status or language with the MeSH terms (“Massage” or “Tuina” or “Zone Therapy”) and (“gastrointestinal dysfunction” or “gastrointestinal motility”).

### 2.4 Selection of studies and data extraction

**Figure 1:**
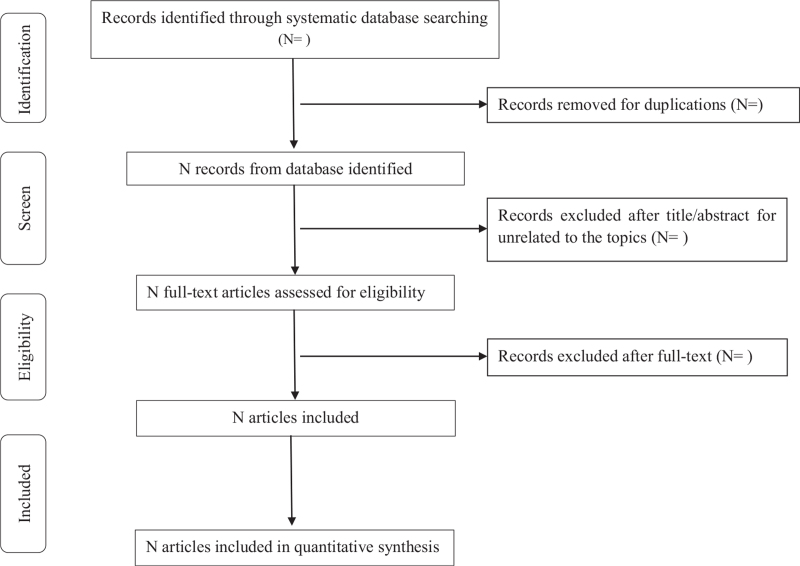
Flow chart of study selection.

Two authors (WYL and XJB) will retrieve and organize all potentially relevant articles in the Endnote X9 (Clarivate Analytics). Then 2 authors (WYL and XJB) will independently screen the titles and abstracts and retrieve the full texts of all potentially eligible studies. Two authors (WYL and XJB) will independently examine the full-text articles for compliance with the inclusion criteria. For the included studies, 2 authors (WYL and XJB) will independently extract data by a standard data extraction table designed according to Cochrane guidelines, including publication of year, author, participants, intervention, control, duration of intervention, outcomes, and methodological characteristics. If there is any disagreement on the selection of articles and the process of data extraction, they will be discussed with the third author (LZX). The study selection procedure will be shown in a preferred reporting items for systematic reviews and meta-analyses statement flow chart (Fig. 1).

### 2.5 Assessment of the risk of bias

Two authors (WYL and XJB) will independently assess the risk of bias using the Cochrane tool of risk of bias (V.5.1.0), including random sequence generation (selection bias), allocation concealment (selection bias), blinding (performance bias and detection bias), incomplete outcome data (attrition bias), selective outcome reporting (reporting bias), and other bias. The judgments of evaluated domains will include high, low, and unclear. Disagreements will be resolved by discussion by arbiter (BR).

### 2.6 Assessment of reporting biases

In view of the difficulty in detecting and correcting for publication bias and other reporting bias, we will minimize their potential impact by ensuring a comprehensive search for included studies and by being aware of duplicated data. Moreover, we will use funnel plots to explore the possibility of a small study effect, where there are sufficient studies. If asymmetry of funnel plots suggest possible small study effects, we will cautiously explain the results.^[14,15]^

### 2.7 Assessment of heterogeneity

According to Cochrane Handbook for Systematic Reviews of Interventions, we will use the I^2^ statistic to examine heterogeneity for quantifying inconsistency in all included studies. Where I^2^ value is greater than 50%, substantial heterogeneity will be indicated.

### 2.8 Data synthesis and statistical analysis

Based on the guideline developed by Cochrane Collaboration, we will perform statistical analysis using RevMan 5.3 software (Cochrane). We will express continuous variables as mean difference with 95% confidence intervals. For categorical variables, we will calculate risk ratios with 95% confidence intervals. In this review, we will include all parallel-designed studies. For cross-over trials, we will include and analyze only the first treatment period data. For studies with multiple control groups, the unit of analysis will be used to each of all control groups. For insufficient or missing data, we will contact the authors by e-mail or phone as much as possible. All analysis will be performed based on intent-to-treat principle. We will conduct a fixed-effect model when I^2^ < 50% or a random-effect model will be performed.

### 2.9 Subgroup analysis and sensitivity analysis

Considering the differences of methodological quality, types of massage and race/ethnicity, we will performed subgroup analysis. To assess the robustness of data analysis, sensitivity analysis will be conducted.

### 2.10 Confidence in cumulative evidence

In this study, the level of evidence on outcomes will be assessed using an approach based on the grading of recommendations assessment, development and evaluation.^[16]^ The quality of the body of evidence will be assessed based on 5 factors, including study limitations, effect consistency, imprecision, indirectness, and publication bias. The assessments will be categorized as high, moderate, low, and very low quality.

## 3 Ethics and dissemination

In this study, ethical approval is not required, in consideration of this protocol for a systematic review. There will be no participants recruited, no data gathered from participants. This review will be disseminated by the approach of peer-reviewed publications.

## Author contributions

**Conceptualization:** Yongliang Wang, Jiaben Xu, Rui Bao, Zhaoxian Li.

**Data curation:** Rui Bao, Zhaoxian Li.

**Formal analysis:** Yongliang Wang, Jiaben Xu, Rui Bao.

**Funding acquisition:** Yongliang Wang, Zhaoxian Li.

**Methodology:** Yongliang Wang, Rui Bao.

**Project administration:** Yongliang Wang.

**Software:** Yongliang Wang, Jiaben Xu, Zhaoxian Li.

**Supervision:** Yongliang Wang, Zhaoxian Li.

**Writing – original draft:** Yongliang Wang, Jiaben Xu, Rui Bao, Zhaoxian Li.

**Writing – review & editing:** Yongliang Wang, Jiaben Xu, Rui Bao, Zhaoxian Li.
